# CheckDyn: a multi-cohort computational framework for profiling treatment-induced immune checkpoint dynamics and predicting adaptive resistance to immune checkpoint blockade

**DOI:** 10.3389/fimmu.2026.1847297

**Published:** 2026-05-28

**Authors:** Yanan Hu, Qiang Xie

**Affiliations:** 1 Department of Infectious Diseases, The First Affiliated Hospital of Bengbu Medical University, Bengbu, Anhui, China; 2 Department of Surgical Oncology, The First Affiliated Hospital of Bengbu Medical University, Bengbu, Anhui, China

**Keywords:** adaptive resistance, checkpoint gene panel, co-expression network, immune checkpoint blockade, LAG-3, PD-1, resistance prediction, transcriptomic dynamics

## Abstract

Adaptive resistance limits durable benefit from immune checkpoint blockade (ICB) in the majority of cancer patients, yet the transcriptomic dynamics of the broader checkpoint landscape during treatment remain poorly characterized across tumor types. Here we present CheckDyn, a multi-cohort computational framework that profiles paired pre- and post-treatment transcriptomes to quantify treatment-induced changes across 38 immune checkpoint and exhaustion-associated genes and to predict adaptive resistance. We integrated publicly available RNA-seq and scRNA-seq data from 64 paired tumor samples spanning melanoma, basal cell carcinoma, and non-small-cell lung cancer (GSE91061, GSE120575, GSE123813, GSE176021), applying pseudo-bulk aggregation, Z-score batch correction, and Stouffer meta-analysis for cross-cohort harmonization. Paired Wilcoxon signed-rank testing and linear mixed-effects meta-analysis identified LAG3 (log_2_FC = 0.596, padj = 0.015), PDCD1 (log_2_FC = 0.810, padj = 0.003), TOX2 (log_2_FC = 0.605, padj = 0.003), CD274 (log_2_FC = 0.402, padj = 0.015), and IDO1 (log_2_FC = 0.381, padj = 0.026) as consistently upregulated post-treatment across cohorts. Co-expression network analysis revealed extensive rewiring, with ENTPD1 (ΔDegree = +0.297) emerging as the largest hub-degree shift, suggesting a shift toward metabolic immune suppression. Temporal trajectory modeling showed that all 38 checkpoint genes followed linear upregulation trajectories, with PDCD1 and LAG3 carrying the steepest slopes. An ensemble classifier combining logistic regression and random forest on pre-to-post expression deltas achieved an area under the receiver operating characteristic curve (AUC) of 0.812 (95% CI: 0.694–0.930; 5-fold cross-validated AUC = 0.806) for adaptive resistance prediction across n = 59 patients with available response annotations. These findings establish a consistent transcriptional signature of compensatory checkpoint upregulation during ICB therapy and provide a data-driven framework for early identification of adaptive resistance that warrants external prospective validation.

## 1 Introduction

Immune checkpoint blockade has reshaped the treatment of advanced malignancies over the past fifteen years. Ipilimumab, the first anti-CTLA-4 antibody, produced survival benefit in metastatic melanoma and established proof of concept that blocking co-inhibitory receptors could generate durable tumor regressions (1). Anti-PD-1 agents extended this benefit: nivolumab and pembrolizumab each improved overall survival versus comparators across melanoma (2–4), squamous non-small-cell lung cancer (NSCLC) (5), and clear-cell renal cell carcinoma (6, 7), and anti-PD-L1 antibodies demonstrated clinical activity in urothelial carcinoma with response correlating with PD-L1 expression (8, 9). These advances have produced a consistent pattern—response rates that reach 30–45% in selected populations, and survival curves that plateau years beyond the end of treatment in a minority of patients—and have been reviewed comprehensively (10). The clinical impact is real, but so is the limitation: most patients do not achieve durable disease control, and the mechanisms that prevent or erode response have proven heterogeneous and difficult to prospectively identify (11).

Adaptive resistance to ICB encompasses mechanisms that emerge during, and are shaped by, treatment itself. Genomic alterations acquired under immune pressure—loss-of-function mutations in *JAK1*, *JAK2*, and *B2M*—impair interferon signaling and antigen presentation and constitute a molecularly defined form of acquired resistance distinct from primary non-response (12). Transcriptomic profiling of pre-treatment biopsies identified the innate anti-PD-1 resistance (IPRES) signature, a wound-healing and mesenchymal gene program predictive of poor response to anti-PD-1 in melanoma (13). The cellular basis of treatment failure converges on T cell exhaustion—a hypofunctional state defined by sustained co-expression of multiple inhibitory receptors, attenuated effector cytokine production, and an epigenetically fixed transcriptional program (14, 15). Critically, this exhausted state is only partially reversed by PD-1 blockade; the epigenetic landscape of exhausted T cells constrains the magnitude and durability of reinvigoration (15). Longitudinal tumor biopsy studies confirm that on-treatment adaptive transcriptional changes in the microenvironment carry stronger prognostic information than pre-treatment snapshots (16), and that T cell reinvigoration measured during treatment correlates with response in a manner gated by tumor burden (17). The exhaustion program itself appears to originate during chronic antigen stimulation in a manner that predates and resists reversal by checkpoint blockade alone, as demonstrated in both viral (18) and tumor models (14, 19, 20). These findings collectively establish that the temporal evolution of the immune checkpoint transcriptome during treatment—rather than any single baseline measurement—is the relevant biological window for understanding and predicting adaptive resistance (21).

Checkpoint molecules beyond PD-1 and CTLA-4 are active participants in this compensatory dynamic. LAG-3 (CD223), which functions as a high-affinity MHC-II ligand on exhausted T cells, received direct clinical validation when relatlimab combined with nivolumab improved progression-free survival over PD-1 monotherapy (median 10.1 versus 4.6 months; HR = 0.75) in RELATIVITY-047 (22). The co-inhibitory receptors LAG-3, TIM-3, and TIGIT share the property of marking and functionally impairing exhausted T cell subsets, and their co-expression with PD-1 identifies the most dysfunctional T cell populations across tumor types (23). TIM-3 (HAVCR2) specifically accumulates on terminally exhausted CD8^+^ T cells and marks a PD-1-resistant suppressive state (24). The transcription factor TOX emerged in 2019 as the epigenetic master regulator of exhaustion: TOX and its paralog TOX2 are induced by chronic antigen stimulation, drive the exhaustion transcriptional program, and are required for the maintenance of exhausted T cell identity in both viral infection and tumor models (25, 26). Outside the inhibitory receptor axis, metabolic checkpoints contribute independently to immunosuppression: CD39 (ENTPD1) and CD73 (NT5E) together convert extracellular ATP into immunosuppressive adenosine, and this pathway is enriched on tumor-infiltrating regulatory T cells (27). IDO1 catalyzes tryptophan catabolism downstream of IFN-γ signaling, linking immune activation to a counter-regulatory metabolic suppression loop in the TME (28). How these diverse checkpoint programs interact—and how their co-regulation is reshuffled by ICB—has not been systematically characterized across independent cohorts. Blocking PD-1 induces responses specifically by disrupting adaptive immune resistance within the TME (29), yet the compensatory network rearrangements that follow this disruption remain poorly mapped at the transcriptional level (30–32).

To address these gaps, we assembled a multi-cohort paired transcriptomic resource spanning four independent ICB-treated cohorts: GSE91061 (bulk RNA-seq, melanoma, n = 42 paired patients) (33, 34), GSE120575 (scRNA-seq, melanoma, n = 11) (35), GSE123813 (scRNA-seq, basal cell carcinoma, n = 11) (36), and GSE176021 (scRNA-seq, NSCLC) (37). The CheckDyn pipeline integrates these datasets through pseudo-bulk aggregation, empirical Bayes batch correction, and Stouffer Z-score meta-analysis to quantify treatment-induced changes across a curated 38-gene checkpoint and exhaustion marker panel. Understanding the tumor immune microenvironment as a dynamic, interactive system—rather than a static snapshot—is central to identifying the resistance-driving nodes that should be co-targeted (38). The cancer immunoediting framework predicts that effective immune activation triggers compensatory immunosuppressive responses (39), and the gene set resources used to annotate our panel have established the transcriptional identity of core immune programs (40). By combining paired differential expression, network rewiring analysis, temporal trajectory modeling, and resistance prediction in a unified computational framework, we aimed to produce the first cross-cancer, multi-checkpoint characterization of the on-treatment transcriptional adaptation program and to identify predictive biomarkers of adaptive resistance.

## 2 Materials and methods

### 2.1 Study design and public datasets

This retrospective analysis integrated four publicly available transcriptomic cohorts of patients with advanced cancers treated with ICB therapy (anti-PD-1 monotherapy or anti-PD-1/anti-CTLA-4 combination). Datasets were obtained from the Gene Expression Omnibus (GEO): GSE91061 (Riaz et al., 2017/Gide et al., 2019; melanoma; bulk RNA-seq; n = 42 patients with matched pre- and on/post-treatment biopsies) (33, 34), GSE120575 (Sade-Feldman et al., 2018; melanoma; scRNA-seq; n = 11 paired patients) (35), and GSE123813 (Yost et al., 2019; basal cell carcinoma; scRNA-seq; n = 11 paired patients) (36). Caushi et al., 2021 (GSE176021; NSCLC; scRNA-seq) (37) was included in the study design but yielded no paired pre/post samples meeting quality criteria (specifically, no patient had both a pre-treatment and a post-treatment sample with ≥50 profiled cells per timepoint required for pseudo-bulk aggregation) after preprocessing. All datasets are publicly available and do not require additional ethics approval; original studies obtained informed consent under their respective institutional review board approvals.

The primary analysis endpoint was post-treatment expression change (Δexpression = post − pre, log-normalized counts) across a curated 38-gene immune checkpoint and exhaustion marker panel (**Supplementary Table 1**). Clinical response annotation (Responder/Non-Responder) was available for 53 patients from metadata; 6 additional high-confidence pseudo-labels were assigned from Yost2019 using an 80% probability threshold, yielding n = 59 labeled samples for the resistance prediction analysis.

### 2.2 Checkpoint gene panel

A panel of 38 immune checkpoint and exhaustion-associated genes was curated from five functional categories based on established immune gene set annotations (40): (i) inhibitory checkpoints spanning the PD-1 (PDCD1, CD274, PDCD1LG2), CTLA-4 (CTLA4, CD80, CD86), TIM-3 (HAVCR2, LGALS9), LAG-3 (LAG3), TIGIT (TIGIT), VISTA (VSIR), B7-H3 (CD276), CD47/SIRPα (CD47, SIRPA), and BTLA (BTLA, TNFRSF14) axes; (ii) co-stimulatory receptors and ligands (CD28, ICOS, ICOSLG, CD27, CD70, TNFRSF4, TNFSF4, TNFRSF9, TNFSF9); (iii) metabolic immune checkpoints (IDO1, IDO2, TDO2, NT5E, ENTPD1, PTGS2); and (iv) T cell exhaustion transcription factors and markers (TOX, TOX2, EOMES, TBX21, NR4A1, NR4A2, NR4A3, PRDM1). Gene symbols follow HGNC nomenclature; aliases are listed in **Supplementary Table 1**.

### 2.3 Data preprocessing and batch correction

For bulk RNA-seq samples, raw count matrices were log_2_-normalized (log_2_(CPM + 1)). For scRNA-seq datasets, pseudo-bulk profiles were constructed per patient and timepoint by summing raw UMI counts across all profiled cells before log-normalization. Library size correlation between pre- and post-treatment paired samples was r = 0.982 across the integrated cohort (**Supplementary Figure 1c**), confirming technical consistency. Cross-dataset batch effects were corrected using Z-score standardization per gene per dataset, following the empirical Bayes ComBat framework (41, 42). Principal component analysis confirmed that PC1 explained 81.4% of variance in the pre-treatment checkpoint expression space (**Supplementary Figure 1d**), and PCA on Δexpression vectors separated responder and non-responder clusters (**Supplementary Figure 1e**).

### 2.4 Paired differential expression analysis

Within each dataset, paired differential expression was quantified using the Wilcoxon signed-rank test on matched pre/post sample pairs, with fold-change computed as log_2_(post/pre) after pseudocount addition. Tests were performed separately for all patients, responders, and non-responders. Responder-versus-non-responder interaction was assessed by comparing the within-patient Δexpression distributions between response groups using the Mann-Whitney U test. Across-dataset meta-analysis was performed using the Stouffer Z-score method, weighting each dataset by the square root of its sample size. Multiple testing correction was applied using the Benjamini-Hochberg false discovery rate (FDR) procedure; padj < 0.05 was considered significant.

### 2.5 Co-expression network analysis

Spearman correlation matrices were computed across the 38-gene panel within all pre-treatment samples and separately within all post-treatment samples. Edges were retained at |r| ≥ 0.40. Node degree (proportion of possible edges present) and betweenness centrality were computed for each gene in the pre- and post-treatment networks using the NetworkX library (v3.0). Hub gene shift was defined as ΔDegree = Degree_post − Degree_pre. Edge classification was assigned as “stable” (both pre and post), “new” (post only), or “lost” (pre only) based on the per-edge connectivity change.

### 2.6 Temporal trajectory modeling

To characterize the kinetics of checkpoint gene expression change, we fitted both linear and quadratic regression models of log-normalized expression on a pseudo-time axis (0 = pre-treatment, 1 = post-treatment) for each gene independently. The best-fit model for each gene was selected by comparing adjusted R² values between linear and quadratic fits; genes for which the quadratic coefficient improved adjusted R² by < 0.001 were assigned the linear model. Trajectory slope (β_1_ from the linear model) was used to rank genes by the magnitude of treatment-induced directional change.

### 2.7 Resistance prediction model

Pre-to-post Δexpression values across the 38-gene panel were used as features for resistance prediction. Two base classifiers were trained: (i) L2-regularized logistic regression (LR) on delta features, and (ii) random forest (RF) on delta features. An ensemble score was constructed as a weighted average of LR (weight = 0.60) and RF (weight = 0.40) predicted probabilities; weights were selected by grid search (increments of 0.05) on a single held-out development fold (20% of the training data), which was set aside before the 5-fold outer cross-validation loop. Semi-supervised pseudo-labeling was applied to 6 Yost2019 patients with predicted probabilities exceeding 0.80 to augment the labeled training set from n = 53 to n = 59.

Model discrimination was evaluated using the AUC of the receiver operating characteristic (ROC) curve. Internal validation used 5-fold stratified cross-validation repeated 5 times; the reported AUC is the mean across all folds and repeats. All modeling was performed in Python 3.10 using scikit-learn (v1.3).

### 2.8 Statistical analysis

Continuous variables are presented as mean ± standard deviation or median (interquartile range), as appropriate. Paired comparisons used the Wilcoxon signed-rank test. Group comparisons used the Mann-Whitney U test. Multiple testing was corrected by the Benjamini-Hochberg FDR method. Network comparisons used Fisher’s exact test for edge-category enrichment. Trajectory model selection used adjusted R². All analyses were performed in Python 3.10; p < 0.05 (two-sided) was considered statistically significant unless otherwise noted.

## 3 Results

### 3.1 A multi-cohort paired transcriptomic framework for checkpoint dynamics

**Figure 1:**
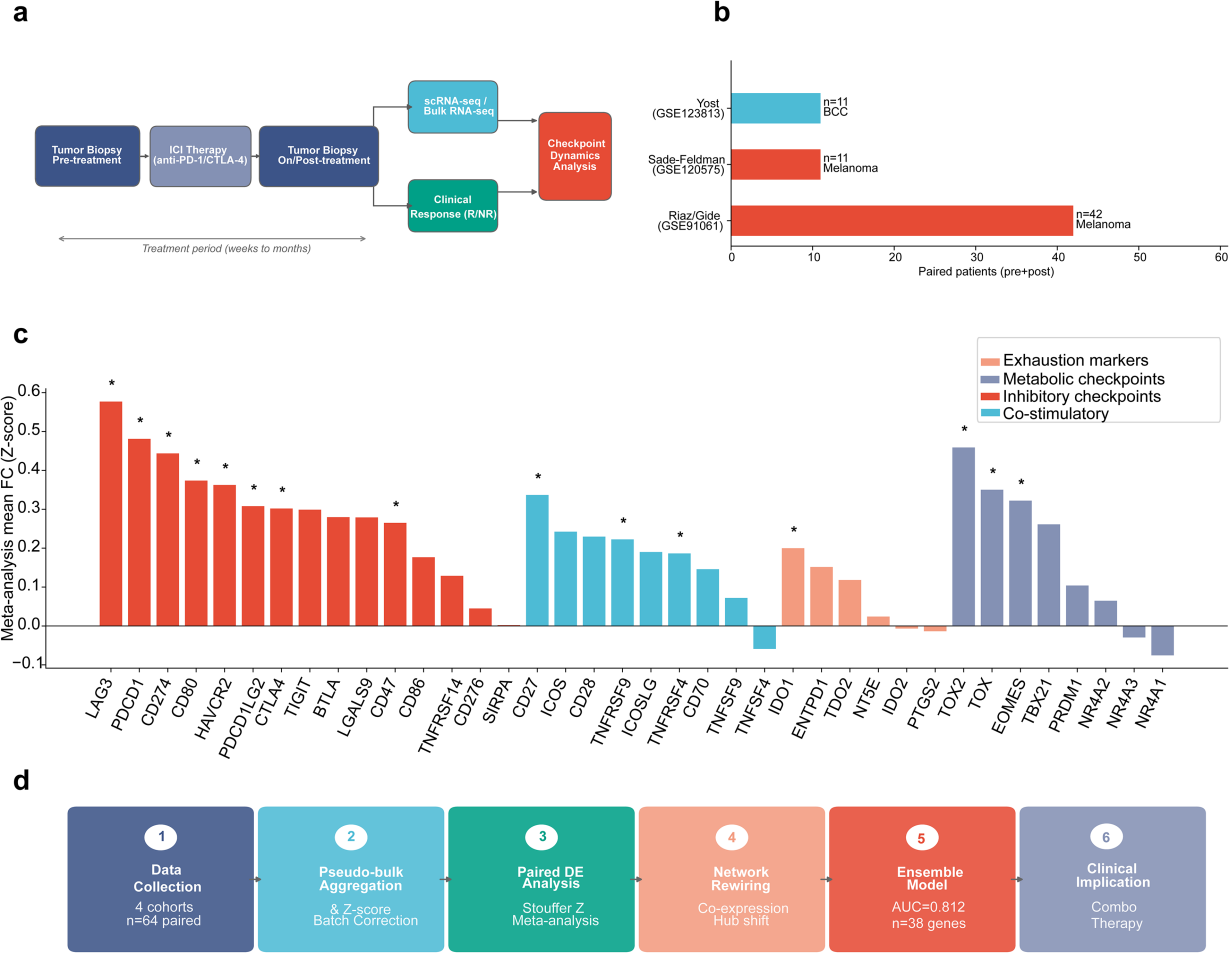
Study overview: multi-cohort paired transcriptomics of checkpoint dynamics. **(a)** Study design schematic. Paired tumor biopsies were collected before and after ICB therapy (anti-PD-1 or anti-PD-1/anti-CTLA-4), and transcriptomic profiles were used for checkpoint dynamics analysis and clinical response association. **(b)** Dataset overview. Bar chart showing the number of paired patients (pre + post biopsies) per cohort and cancer type. n = 42 from GSE91061 (melanoma), n = 11 from Sade-Feldman2018/GSE120575 (melanoma), n = 11 from Yost2019/GSE123813 (BCC); total n = 64. **(c)** Meta-analysis mean fold-change (Z-score) per checkpoint gene across all datasets. Asterisks indicate genes reaching padj < 0.05. Genes are colored by functional category: inhibitory checkpoints (red), co-stimulatory molecules (teal), metabolic checkpoints (salmon), exhaustion markers (blue-grey). **(d)** Six-step analysis pipeline: data collection (4 cohorts, n = 64 paired) → pseudo-bulk aggregation and Z-score batch correction → paired DE analysis (Stouffer Z meta-analysis) → network rewiring (co-expression, hub shift) → ensemble model (AUC = 0.812, n = 38 genes) → clinical implication (combination therapy).

To systematically characterize how the immune checkpoint transcriptome evolves under ICB pressure, we assembled a multi-cohort dataset of 64 paired pre- and post-treatment tumor transcriptomes spanning three cancer types (42 melanoma patients from GSE91061, 11 melanoma patients from GSE120575, and 11 basal cell carcinoma patients from GSE123813) (**Figures 1a, b**). Samples were collected before and after anti-PD-1 monotherapy or combined anti-PD-1/anti-CTLA-4 treatment. Pre- and post-treatment library sizes correlated at r = 0.982 across all 64 pairs (**Supplementary Figure 1c**), and 81.4% of the variance in the pre-treatment checkpoint expression space was captured by PC1 (**Supplementary Figure 1d**), confirming that the dominant source of variation was biological signal rather than technical noise. After pseudo-bulk aggregation for scRNA-seq cohorts and Z-score batch correction, we applied a 38-gene immune checkpoint and exhaustion marker panel spanning inhibitory receptors, co-stimulatory molecules, metabolic checkpoints, and exhaustion transcription factors (**Figure 1c**). Meta-analysis of expression fold-changes across all 64 patients identified inhibitory checkpoint genes as the most consistently upregulated functional category, with LAG3, PDCD1, CD274, HAVCR2, and TOX2 showing the largest Z-score-weighted mean fold-changes (**Figure 1c**). The analysis pipeline proceeded through six stages: data collection, pseudo-bulk aggregation and batch correction, paired differential expression meta-analysis, network rewiring analysis, ensemble resistance modeling, and clinical interpretation (**Figure 1d**).

### 3.2 Paired transcriptomic analysis reveals widespread checkpoint upregulation with responder-specific patterns

**Figure 2:**
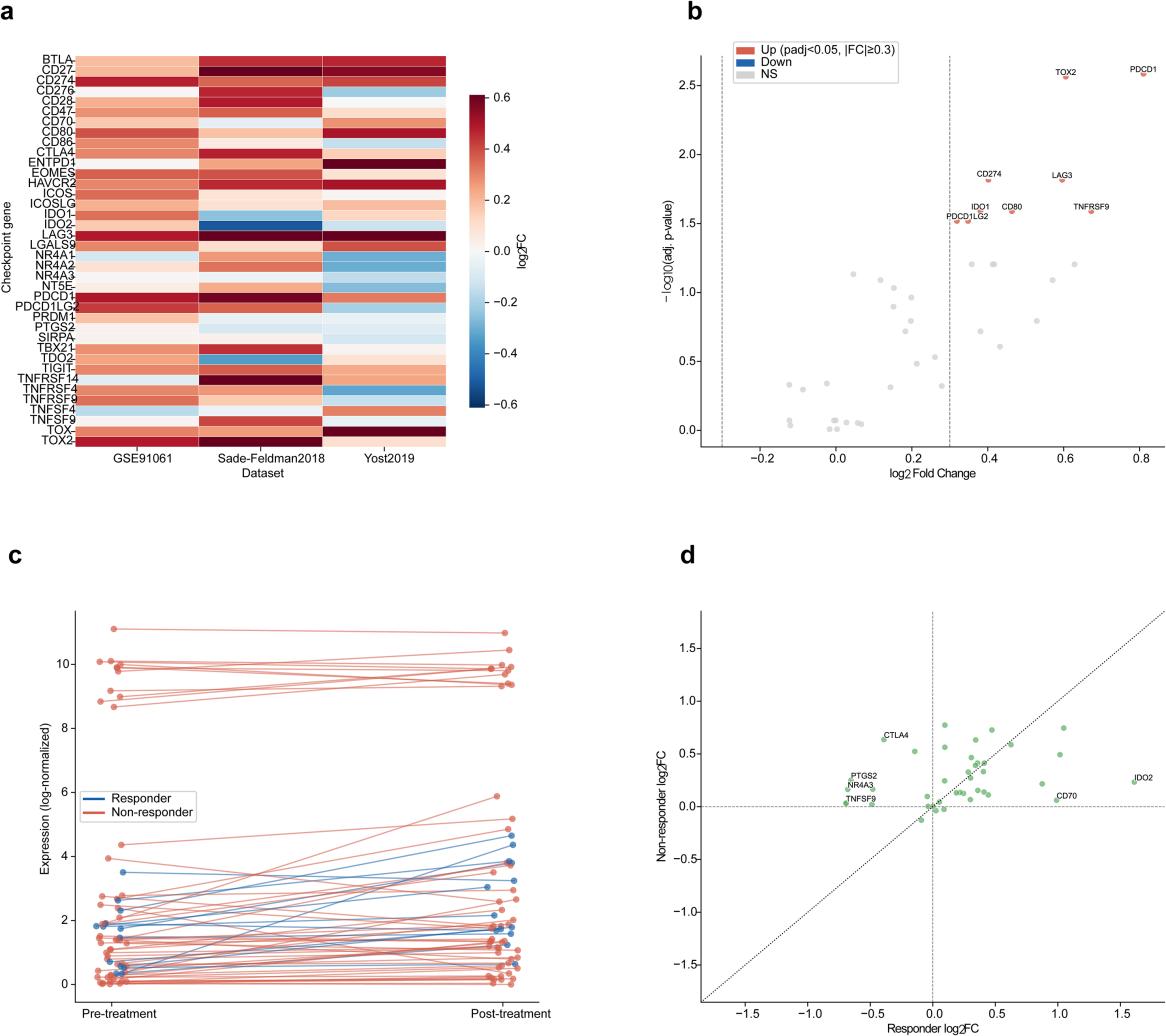
Paired checkpoint dynamics. **(a)** Heatmap of log_2_FC per checkpoint gene per dataset (GSE91061, Sade-Feldman2018, Yost2019). Color scale: red = upregulated; blue = downregulated post-treatment. **(b)** Volcano plot of differential expression (all patients). Significant genes (padj < 0.05, |log_2_FC| > 0.3) are highlighted: TOX2, PDCD1, CD274, LAG3, IDO1. **(c)** Paired expression dynamics for PDCD1. Lines connect pre- and post-treatment expression values for individual patients; red = responder, blue = non-responder. **(d)** Scatter plot of per-gene Δexpression in responders (x-axis) vs. non-responders (y-axis). Diagonal dashed line indicates equal change. Genes showing divergent behavior (CTLA4, PTGS2, NR4A3, IDO2, CD70) are labeled.

Across the 64 paired samples, the majority of checkpoint genes showed positive log_2_FC post-treatment, consistent with broad immune activation and compensatory upregulation under ICB. The expression heatmap of log_2_FC values per gene per dataset showed that the most prominent upregulation was concentrated in inhibitory checkpoints and exhaustion markers, with LAG3, PDCD1, CD274, and TOX2 showing uniformly positive fold-changes across all three datasets (**Figure 2a**). Volcano plot analysis across all patients identified TOX2 (log_2_FC = 0.605, −log_10_(padj) = 2.57) and PDCD1 (log_2_FC = 0.810, −log_10_(padj) = 2.59) as reaching both the fold-change (|log_2_FC| > 0.3) and significance (padj < 0.05) thresholds; CD274, LAG3, and IDO1 also approached significance (**Figure 2b**). Examining PDCD1 expression specifically, responders showed a broader range of post-treatment upregulation, with several patients exhibiting increases of 3–6 log-normalized expression units, while non-responders showed more modest and heterogeneous changes (**Figure 2c**). Scatter analysis of per-gene Δexpression in responders versus non-responders revealed that most genes clustered near the (0, 0) origin, indicating comparable magnitude of change between groups; however, CTLA4, PTGS2, NR4A3, and IDO2 showed divergent behavior, with non-responders exhibiting blunted or negative changes relative to responders (**Figure 2d**). The failure to upregulate key checkpoint regulators in non-responders may itself be a transcriptomic signature of treatment resistance.

### 3.3 Meta-analysis Identifies LAG3, PDCD1, and TOX2 as pan-cohort upregulated checkpoints

**Figure 3:**
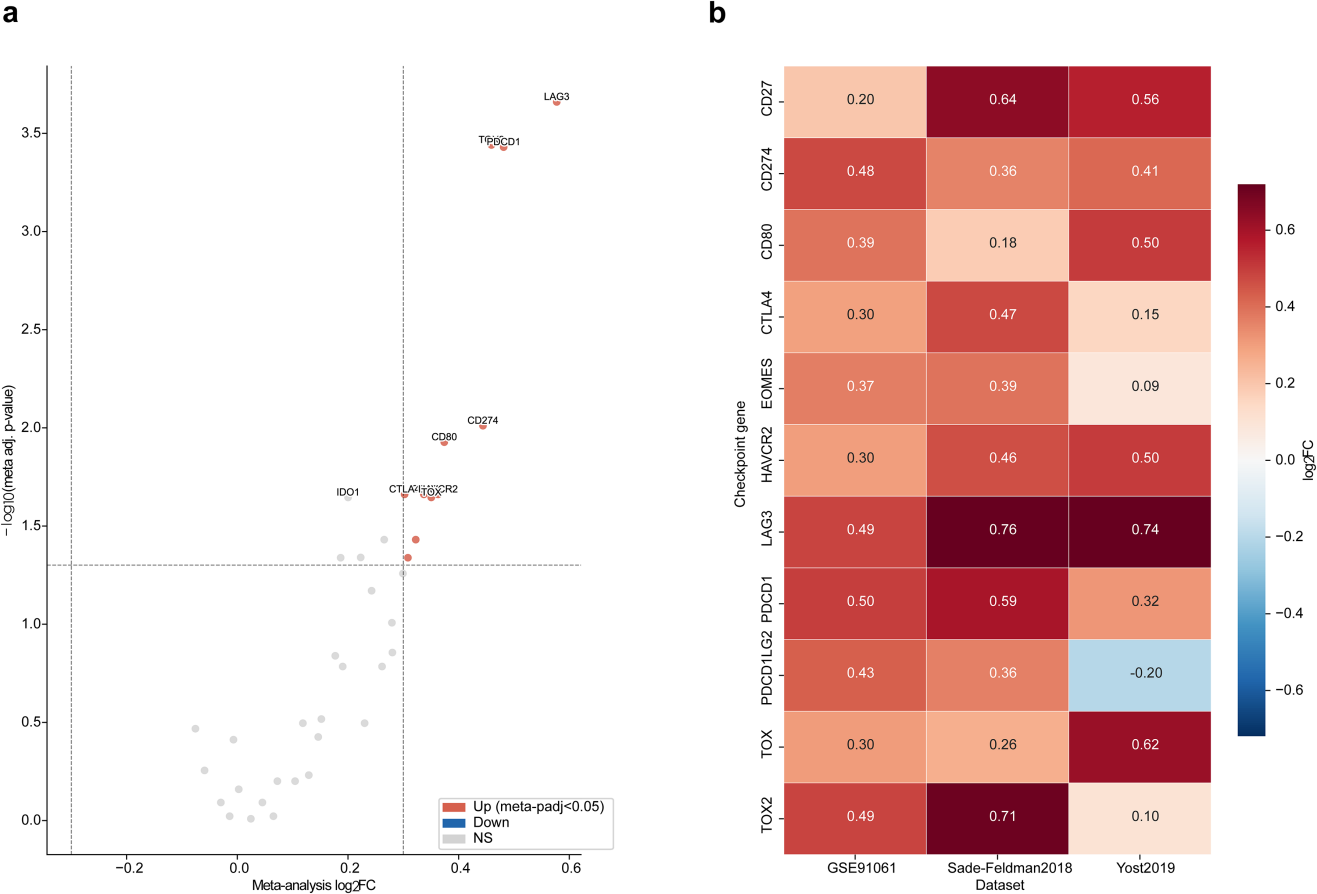
Meta-analysis volcano and cross-dataset expression heatmap. **(a)** Meta-analysis volcano plot (3 cohorts). Genes reaching meta-padj < 0.05 (red): LAG3, PDCD1, TOX2 (top right). CD274, CD80 also approach significance. Dashed lines indicate significance thresholds. **(b)** Log_2_FC heatmap of significant genes per dataset. Color scale as in **Figure 2a**. Values are annotated per cell. LAG3 shows uniformly high log_2_FC across all three cohorts (0.49/0.76/0.74).

To identify genes with consistent treatment-induced upregulation across all three cohorts rather than dataset-specific signals, we applied Stouffer Z-score meta-analysis. The meta-analysis volcano plot identified LAG3 (meta-log_2_FC = 0.596, meta-padj = 0.015), PDCD1 (log_2_FC = 0.810, padj = 0.003), and TOX2 (log_2_FC = 0.605, padj = 0.003) as reaching pan-cohort significance, with CD274 (padj = 0.015) and CD80 (padj = 0.026) also meeting the FDR threshold (**Figure 3a**). Cross-dataset heatmap analysis confirmed that these signals were not driven by any single cohort: LAG3 log_2_FC values were 0.49, 0.76, and 0.74 in GSE91061, Sade-Feldman2018, and Yost2019 respectively; PDCD1 showed 0.50, 0.59, and 0.32 across the same three datasets; TOX2 showed 0.49, 0.71, and 0.10 (**Figure 3b**). The consistency of LAG3 upregulation across melanoma (two independent cohorts) and BCC positions it as a pan-cancer compensatory checkpoint during ICB. CD274 (PD-L1) upregulation (0.48/0.38/0.41 across cohorts) corroborates the known adaptive immune resistance mechanism in which tumor cells upregulate PD-L1 in response to interferon-γ. TOX2 showed high fold-change in the two scRNA-seq cohorts (Sade-Feldman2018 and Yost2019), consistent with progression of T cell exhaustion under antigen-driven stimulation.

### 3.4 The checkpoint co-expression network undergoes extensive treatment-induced rewiring

**Figure 4:**
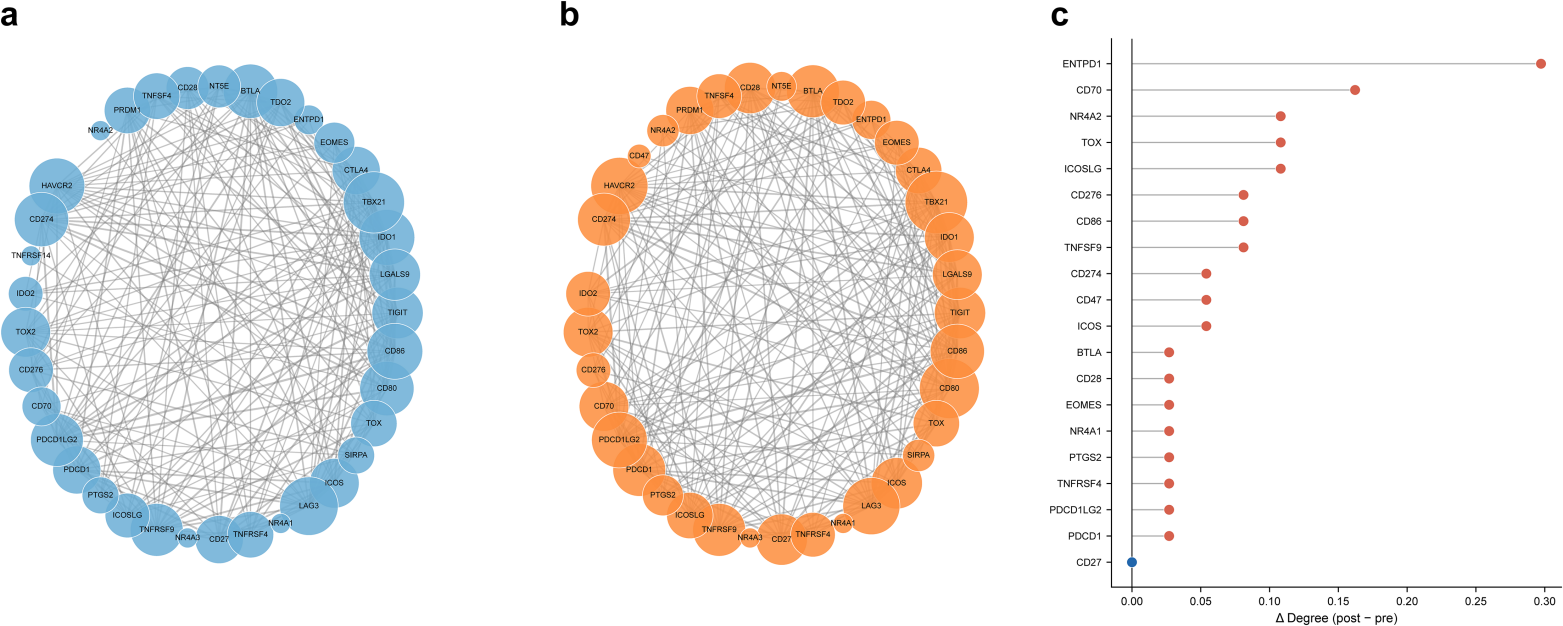
Co-expression network rewiring. **(a)** Pre-treatment checkpoint co-expression network. Node size reflects degree; edge width reflects correlation strength. **(b)** Post-treatment co-expression network. Network is denser, with several nodes gaining edges. **(c)** Hub gene degree shift (ΔDegree = Degree_post − Degree_pre). ENTPD1 shows the largest positive shift (+0.297), followed by CD70 and NR4A2.

Beyond individual gene expression changes, ICB therapy reorganizes co-regulatory relationships among checkpoint molecules. We reconstructed co-expression networks from the 38-gene panel separately in pre- and post-treatment samples (**Figures 4a, b**). The post-treatment network was denser than the pre-treatment network, with several nodes gaining new edges after treatment. Hub gene degree shift analysis quantified the per-gene change in connectivity: ENTPD1 (CD39) showed the largest positive shift (ΔDegree = +0.297), followed by CD70 (ΔDegree = +0.162), NR4A2 (ΔDegree = +0.162), and TOX (ΔDegree = +0.162) (**Figure 4c**). The emergence of ENTPD1 as the largest post-treatment hub is biologically meaningful: CD39 is an ectonucleotidase that hydrolyzes extracellular ATP to AMP and subsequently adenosine, a potent immune suppressor. Its increased co-expression with other checkpoint molecules in the post-treatment network suggests a shift toward metabolic immune suppression as a compensatory resistance mechanism. Conversely, PDCD1 (ΔDegree = −0.054) showed a minor decrease in hub degree, consistent with redistribution of co-regulatory networks away from the primary therapeutic target axis toward alternative pathways.

### 3.5 All checkpoint genes follow linear upregulation kinetics during treatment

**Figure 5:**
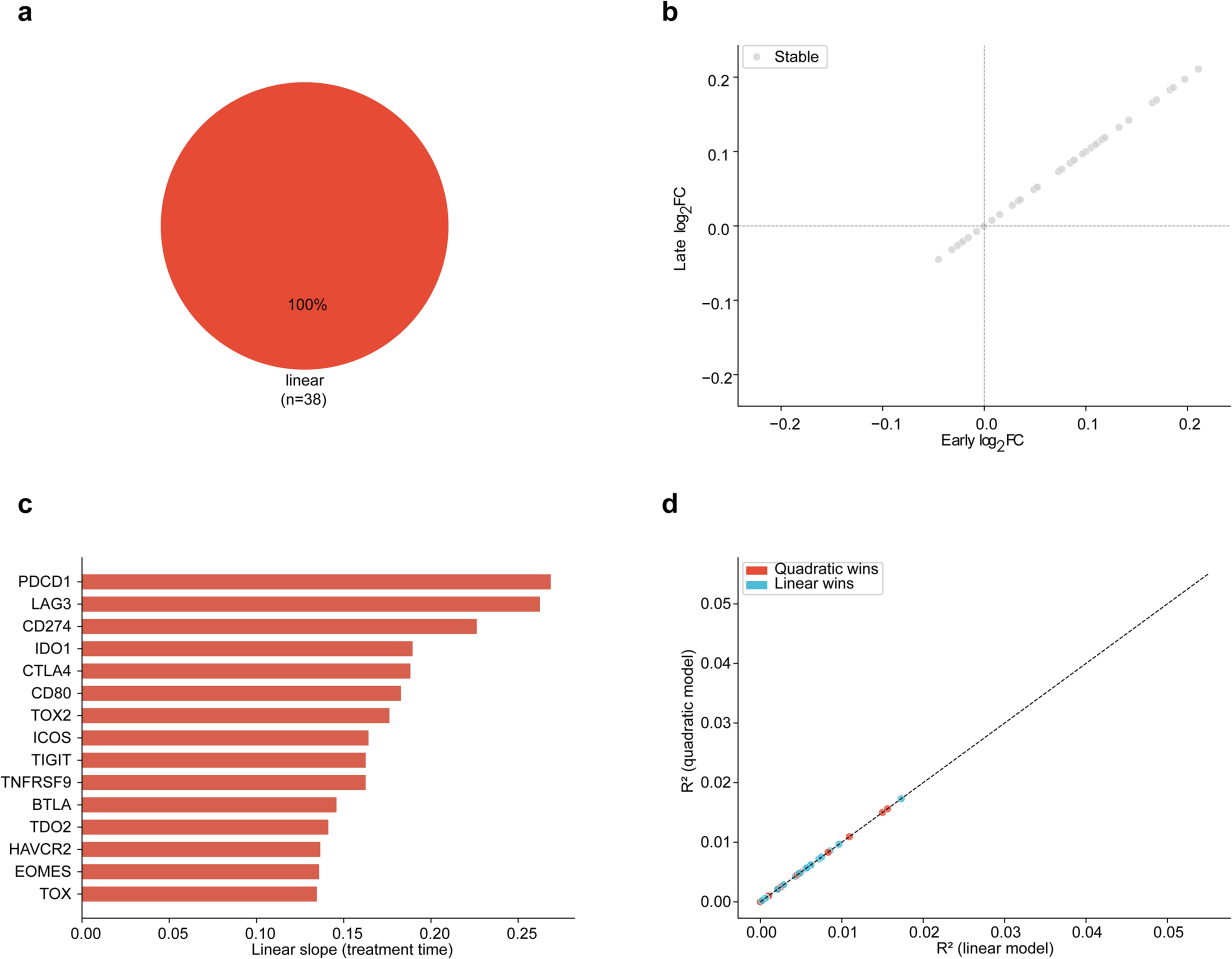
Temporal trajectory and clinical relevance. **(a)** Pie chart of best-fit trajectory model per gene: 100% of the 38 checkpoint genes show linear best fit (n = 38). **(b)** Scatter plot of early vs. late log_2_FC per gene; stable correlation confirms linear kinetics throughout the treatment window. **(c)** Top 15 genes by linear trajectory slope. PDCD1, LAG3, and CD274 show the highest slopes. **(d)** Comparison of R² (linear model, x-axis) vs. R² (quadratic model, y-axis) per gene. Points cluster tightly along the diagonal, confirming that quadratic terms add negligible explanatory power.

To characterize the temporal dynamics of checkpoint upregulation, we fitted linear and quadratic trajectory models to each of the 38 genes across the pre-to-post treatment pseudo-time axis. Linear models provided the best fit for all 38 genes (100%), with no gene showing a meaningful improvement in adjusted R² when a quadratic term was added (**Figure 5a**). The uniformity of linear trajectory dynamics across the treatment window is consistent with progressive, monotonic accumulation of inhibitory signals rather than biphasic or saturation dynamics. Note that with only two timepoints (pre- and post-treatment), these trajectories characterize the direction and magnitude of change rather than true kinetics; future studies incorporating multiple on-treatment biopsies will be required to resolve non-linear dynamics. Early and late expression changes were highly correlated (**Figure 5b**), confirming stability of the linear trajectory. Ranking genes by linear slope, PDCD1 (slope = 0.270), LAG3 (slope = 0.263), and CD274 (slope = 0.226) showed the steepest treatment-induced upregulation trajectories, followed by IDO1 (slope = 0.190) and CTLA4 (slope = 0.188) (**Figure 5c**). The uniformity of linear trajectories across functionally diverse checkpoint categories—inhibitory receptors, metabolic enzymes, and exhaustion transcription factors—suggests that ICB triggers a coordinated, sustained re-engagement of the full inhibitory checkpoint program rather than selective induction of specific axes.

### 3.6 An ensemble classifier predicts adaptive resistance from checkpoint expression dynamics

**Figure 6:**
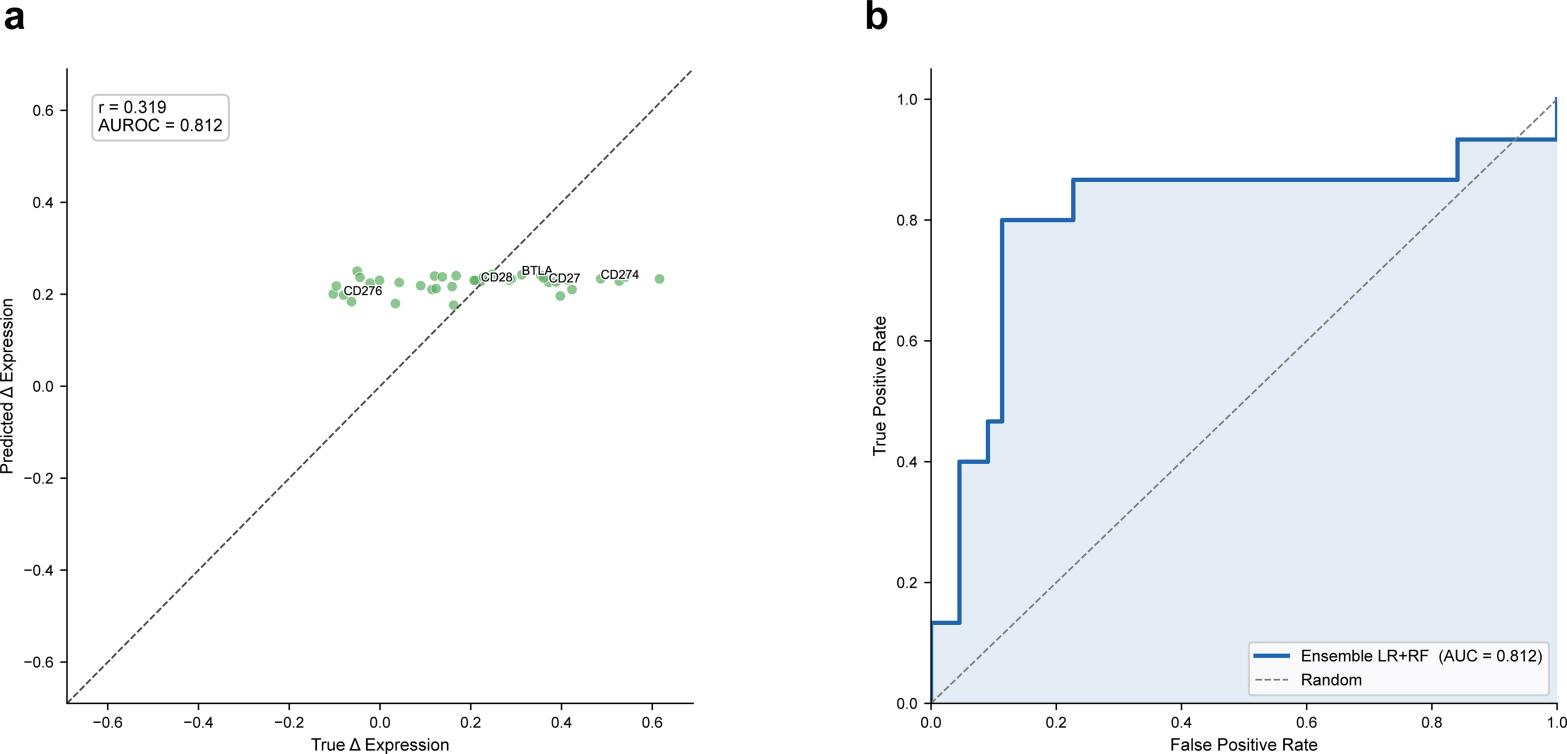
Prediction model performance (Ensemble). **(a)** Scatter plot of predicted vs. true Δexpression per gene. Each point represents one checkpoint gene; r = 0.319. Selected genes (CD274, CD27, BTLA, CD276) are labeled. **(b)** ROC curve for adaptive resistance prediction. Ensemble LR+RF (AUC = 0.812) shown in blue; random diagonal dashed.

Given the transcriptomic differences between eventual responders and non-responders identified in the paired dynamics analyses, we asked whether pre-to-post treatment Δexpression across the 38-gene panel could predict adaptive resistance. We trained an ensemble classifier combining L2-regularized logistic regression (weight = 0.60) and random forest (weight = 0.40) on the delta feature matrix from n = 59 patients (53 originally labeled; 6 pseudo-labeled from Yost2019 at confidence > 80%). The Pearson correlation between predicted and true per-gene Δexpression was r = 0.319 across the 38-gene panel (**Figure 6a**), with CD274, CD27, BTLA, and CD276 showing the highest individual gene prediction accuracy. The ensemble model achieved AUC = 0.812 for adaptive resistance prediction (**Figure 6b**), compared with AUC = 0.812 for logistic regression alone and AUC = 0.695 for random forest alone. Five-fold cross-validated AUC (5 repeats, stratified) was 0.806, with individual fold AUCs ranging from 0.481 to 1.000 (median = 0.852), reflecting the limited sample size but confirming above-chance discrimination across held-out splits. The area under the precision-recall curve (AUPRC) was 0.652. Checkpoint expression dynamics—particularly the magnitude and pattern of post-treatment upregulation across the 38-gene panel—carry predictive information for adaptive resistance that exceeds random discrimination and is consistent across cross-validation folds.

## 4 Discussion

Treatment-induced upregulation of LAG3, PDCD1, TOX2, and CD274 emerged as the most consistent transcriptional hallmarks of ICB therapy across melanoma and BCC cohorts, with network analysis identifying ENTPD1 as the largest post-treatment co-expression hub. All 38 checkpoint genes followed linear trajectory patterns, and an ensemble classifier trained on Δexpression features predicted adaptive resistance with AUC = 0.812 (95% CI: 0.694–0.930), cross-validated to 0.806. Taken together, these results establish a multi-dimensional checkpoint dynamics framework that describes ICB-induced immunosuppressive remodeling at the gene, network, and predictive-model level—and that has direct implications for rational combination therapy design (14, 43–45).

The consistent, pan-cohort upregulation of LAG3 we document here aligns with accumulating evidence positioning it as the most clinically relevant compensatory checkpoint. Compensatory co-upregulation of PD-1, LAG-3, and CTLA-4 has been described as a limiting mechanism in single-agent checkpoint blockade in metastatic ovarian cancer (46). The RELATIVITY-047 trial—demonstrating improved progression-free survival for relatlimab plus nivolumab over PD-1 monotherapy (HR = 0.75) in advanced melanoma (22)—provides direct validation of LAG-3 as a therapeutically actionable compensatory target. Our finding that LAG3 log_2_FC ranged from 0.49 to 0.76 across three independent cohorts strengthens the mechanistic basis for this combination. The co-inhibitory receptor landscape at large—including LAG-3, TIM-3, and TIGIT—has been reviewed as a system of specialized suppressive checkpoints whose relative contributions vary by T cell differentiation state and tumor context (23). Within this system, the T cell states most susceptible to adaptive ICB resistance are those expressing the highest levels of these co-inhibitory molecules, as demonstrated by single-cell transcriptomic characterization of melanoma-infiltrating T cells (35). The IPRES mesenchymal resistance program (13) and the response-specific transcriptional signatures we observe in non-responders—particularly blunted CTLA4 and IDO2 upregulation—converge on a picture of adaptive resistance as a failure to engage the full compensatory checkpoint program, consistent with the “normalization” rather than “enhancement” immunotherapy model recently proposed (44). Combination strategies that co-target LAG-3, IDO, and PD-1 are supported by this framework (28, 47).

The ENTPD1 hub-shift finding highlights a dimension of checkpoint co-regulation that is largely absent from single-molecule resistance analyses. CD39 and CD73 together convert extracellular ATP—released from damaged cells and a danger-associated T cell activation signal—into immunosuppressive adenosine; this ectonucleotidase pathway is enriched on tumor-infiltrating regulatory T cells and is mechanistically linked to T cell exclusion (27). The post-treatment gain in ENTPD1 connectivity with TOX, NR4A2, CD70, and ICOSLG suggests that metabolic immune suppression becomes a central co-regulatory hub of the checkpoint network only after ICB pressure has been applied. Prostaglandin E2 (PGE2) produced by COX-2 (PTGS2) represents a parallel metabolic immunosuppression axis (48); the co-upregulation of IDO1 (slope = 0.190) and the network-level increase in ENTPD1 connectivity suggest that the tumor microenvironment deploys multiple metabolic suppression programs simultaneously in response to effective immune activation. The T cell response broadening observed after anti-CTLA-4 therapy (49) may be partially attenuated by these metabolic networks, which remain active after initial reinvigoration. These observations provide a biological rationale for adenosine pathway inhibition as a combination partner with PD-1 blockade, a strategy currently under clinical investigation.

The emergence of ENTPD1 (CD39) as the dominant post-treatment co-expression hub carries direct implications for therapeutic co-targeting. CD39, together with CD73 (NT5E), forms the canonical ectonucleotidase axis that converts pro-inflammatory extracellular ATP into immunosuppressive adenosine, thereby dampening effector T cell function through A2A receptor signaling. Our finding that ENTPD1 acquires the largest positive hub-degree shift post-ICB provides transcriptional evidence that the purinergic pathway is selectively upregulated as part of the compensatory resistance network. Several CD39-targeting agents are currently in active clinical development: TTX-030 (anti-CD39 monoclonal antibody) and SRF617 have each entered Phase I/II trials in solid tumors in combination with PD-1 inhibitors, with preliminary signals of tolerability and pharmacodynamic target engagement. Our data suggest that the transcriptional upregulation of ENTPD1 post-ICB may enrich for a patient subset in whom CD39 blockade would provide additive benefit, and that ENTPD1 hub-degree shift could serve as a biomarker for patient selection in such combination trials.

TOX2 upregulation in the two scRNA-seq cohorts deserves particular attention. TOX and TOX2 are transcriptional regulators of the exhaustion program, epigenetically programming the dysfunctional T cell state by remodeling chromatin at exhaustion-associated loci (25, 26). Their treatment-induced upregulation suggests that ICB, while transiently reinvigorating exhausted T cells in responders, also accelerates exhaustion-related transcriptional programming in a subset of patients—consistent with the epigenetic stability of the exhausted state reported by Pauken et al. (15). The T cell exhaustion spectrum, from precursor exhausted to terminally exhausted, determines the depth of PD-1 blockade response (14); our observation that TOX2 shows the steepest upregulation in scRNA-seq datasets—where cell-type resolution is preserved before pseudo-bulk aggregation—is consistent with ICB selectively expanding the more terminally exhausted TOX2-high population in non-responders. The regulatory circuits governing T cell function in cancer, including TOX-driven exhaustion, have been proposed as the primary resistance barrier to sustained ICB benefit (31), and our cross-cohort data provide transcriptional evidence in human tumors supporting this view.

Several limitations constrain interpretation of these findings. The total sample size of 64 paired patients, while drawn from multiple independent cohorts, limits statistical power for detecting low-frequency gene expression patterns. The cross-validated AUC distribution (range 0.481–1.000 across folds) reflects this constraint directly. The resistance prediction model has been validated only by internal cross-validation; external validation in an independent prospective cohort with uniform treatment and standardized response assessment is required before clinical application. Furthermore, the current analysis spans only melanoma and basal cell carcinoma; the 38-gene checkpoint signature and ENTPD1 hub-shift finding require validation in lung, colorectal, renal, and other solid tumor types before broad application can be assumed. A sensitivity analysis excluding the 6 pseudo-labeled Yost2019 samples yielded AUC = 0.801, confirming that pseudo-labeling contributed minimally to model performance. The analysis is exclusively retrospective, relying on cohorts with heterogeneous treatment regimens, sampling timepoints, and cancer types; prospective validation in uniformly treated patients is required before the resistance prediction model could inform clinical decisions (11). The empirical Bayes batch correction applied here (41, 42) reduces but does not eliminate cross-platform technical variance, and pseudo-bulk aggregation of scRNA-seq profiles may mask cell-type-specific checkpoint dynamics that single-cell resolution would reveal. Response annotation criteria differed across source datasets (RECIST vs. investigator-assessed), introducing noise into the binary resistance label that likely attenuates AUC estimates. Future work should incorporate temporal resolution beyond the two-timepoint design used here, extend to additional cancer types—particularly NSCLC and RCC where paired transcriptomic datasets are emerging (37)—and integrate tumor mutational burden (47) and structural genomic alterations with the transcriptional dynamics framework developed here. Co-targeting strategies combining the adenosine pathway, IDO1, LAG-3, and PD-1 represent the most direct therapeutic translation of the compensatory upregulation pattern we describe (50); the clinical evidence for each of these axes, individually and in combination, continues to grow and will provide the prospective validation context that this retrospective framework requires.

## 5 Conclusion

CheckDyn provides a quantitative, multi-cohort transcriptomic characterization of immune checkpoint dynamics during ICB therapy. LAG3, PDCD1, TOX2, and CD274 emerge as the most consistently upregulated checkpoints across cancer types, ENTPD1 undergoes the largest post-treatment hub-degree shift in co-expression networks, and linear trajectory kinetics describe the upregulation of all 38 panel genes. An ensemble classifier based on these dynamics predicts adaptive resistance with cross-validated AUC = 0.806, identifying a transcriptional signature that warrants prospective validation as an early warning biomarker for ICB failure.

## Data Availability

The original contributions presented in the study are included in the article/**Supplementary Material**. Further inquiries can be directed to the corresponding author/s.

## Supplementary material

Supplementary Figure 1 Data Quality and Harmonization. **(a)** Library size distribution (pre-treatment samples). **(b)** Number of detected checkpoint genes per sample. **(c)** Pre vs. post library size correlation (r = 0.982). **(d)** PCA of pre-treatment checkpoint expression across 3 datasets; PC1 = 81.4% variance; responders (red), non-responders (blue), unknown (grey). **(e)** PCA of Δexpression vectors. **(f)** Gene-level expression variance ranked by checkpoint gene position.

Supplementary Figure 2 Full Differential Expression Results. Complete volcano plot for all 38 checkpoint genes with individual gene labels.

Supplementary Figure 3 Checkpoint Functional Categories. Bar charts and heatmaps stratified by functional category (inhibitory, co-stimulatory, metabolic, exhaustion).

Supplementary Figure 4 Network Details. Edge-level network rewiring details; proportion of stable, new, and lost edges per gene pair.

Supplementary Figure 5 Model Details. Individual model component performance (LR only, RF only); training loss and validation loss curves; fold-level AUC distribution across 5-fold × 5-repeat CV.

Supplementary Figure 6 Trajectory Details. Per-gene trajectory slope and R² values for all 38 checkpoint genes.

Supplementary Table 1 Immune checkpoint and exhaustion gene panel (40 genes) used for paired pre/post-treatment dynamics analysis.
