# Supplementary figures and images for: CheckDyn: a multi-cohort computational framework for profiling treatment-induced immune checkpoint dynamics and predicting adaptive resistance to immune checkpoint blockade
Source: Front Immunol. 2026 May 28;17:1847297. doi: 10.3389/fimmu.2026.1847297 (PMC13253502; doi:10.3389/fimmu.2026.1847297)

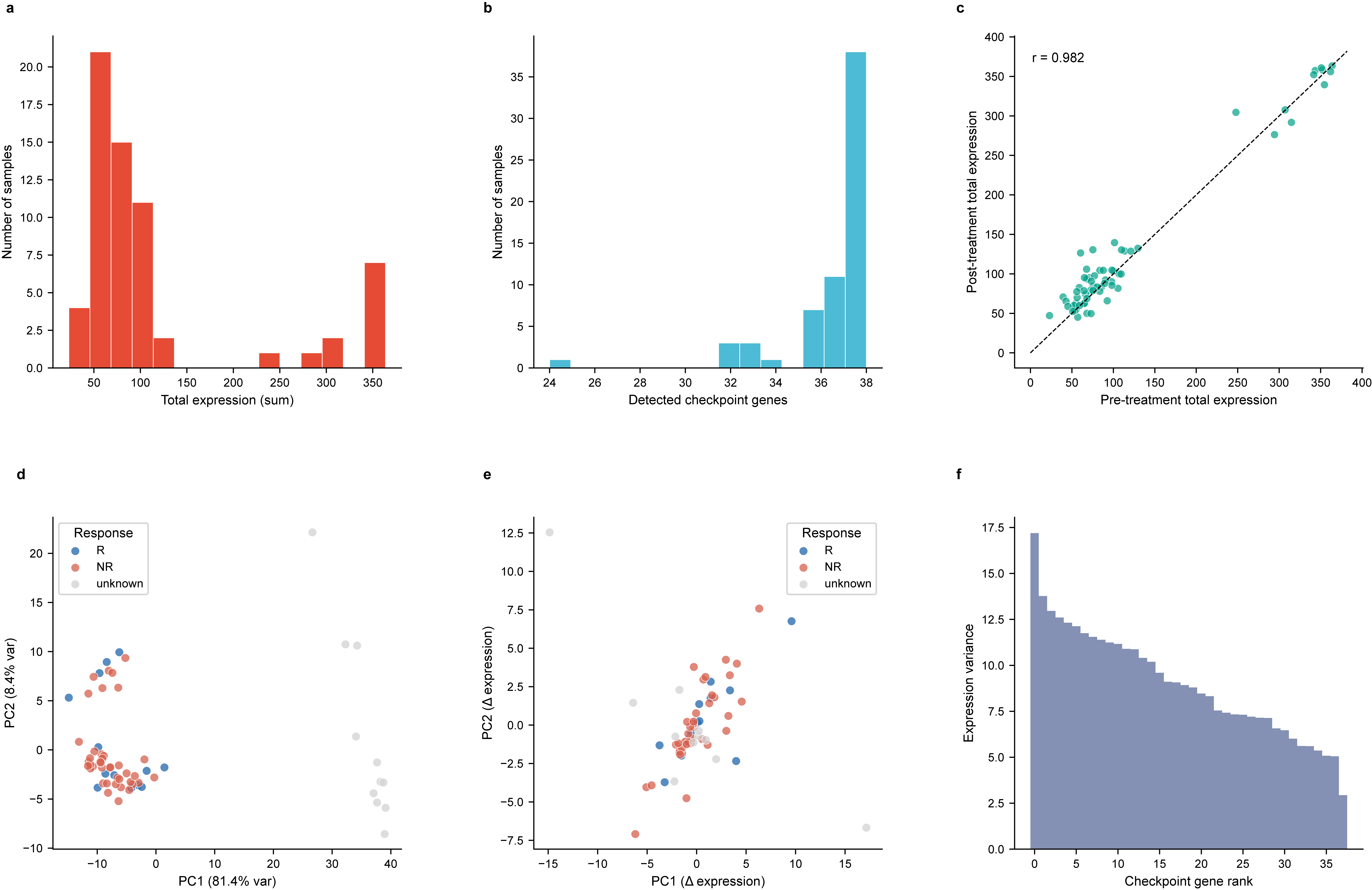

Supplement: Supplementary Figure 1 — Data Quality and Harmonization. (a) Library size distribution (pre-treatment samples). (b) Number of detected checkpoint genes per sample. (c) Pre vs. post library size correlation (r = 0.982). (d) PCA of pre-treatment checkpoint expression across 3 datasets; PC1 = 81.4% variance; responders (red), non-responders (blue), unknown (grey). (e) PCA of Δexpression vectors. (f) Gene-level expression variance ranked by checkpoint gene position. [file Image1.tif]

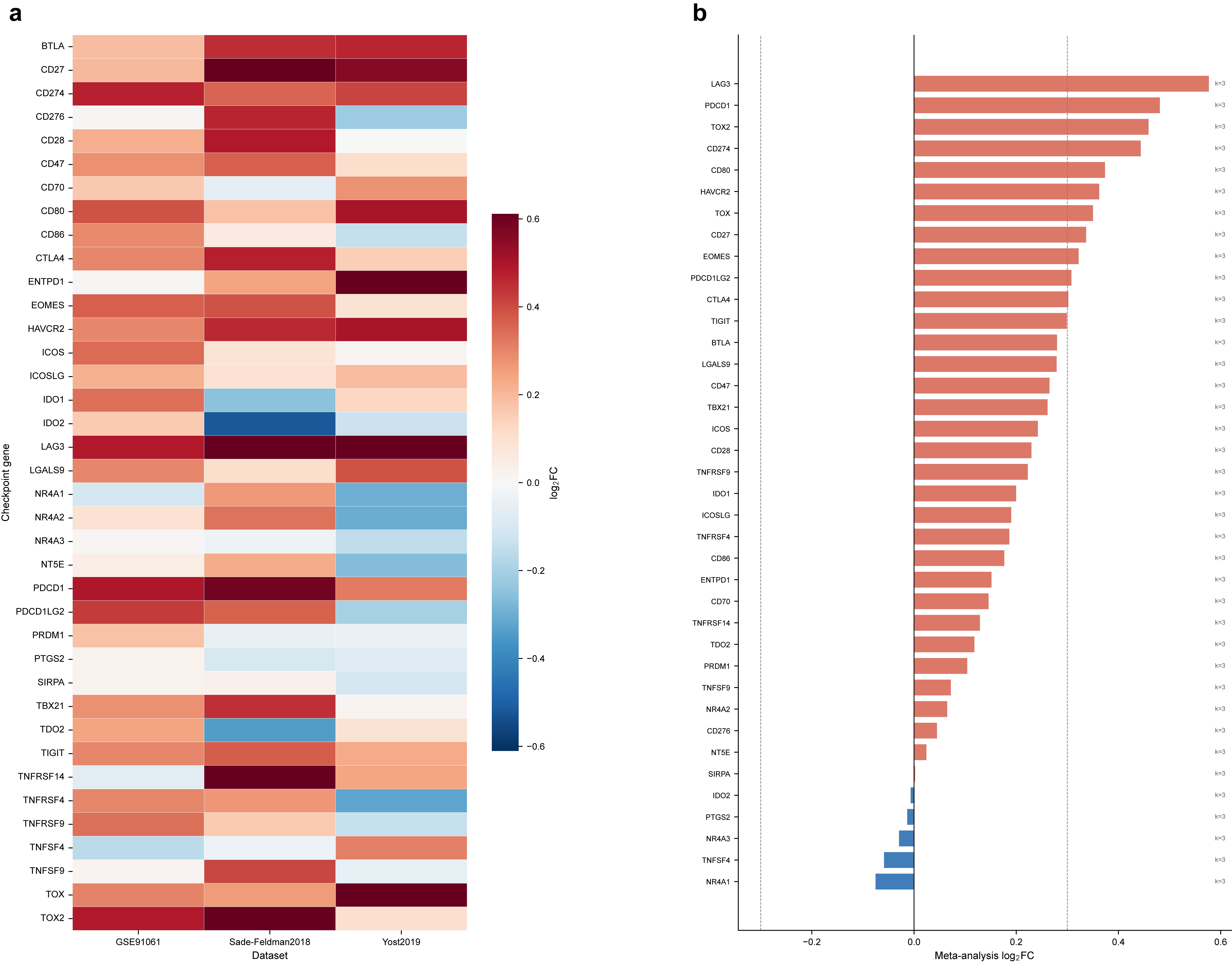

Supplement: Supplementary Figure 2 — Full Differential Expression Results. Complete volcano plot for all 38 checkpoint genes with individual gene labels. [file Image2.tif]

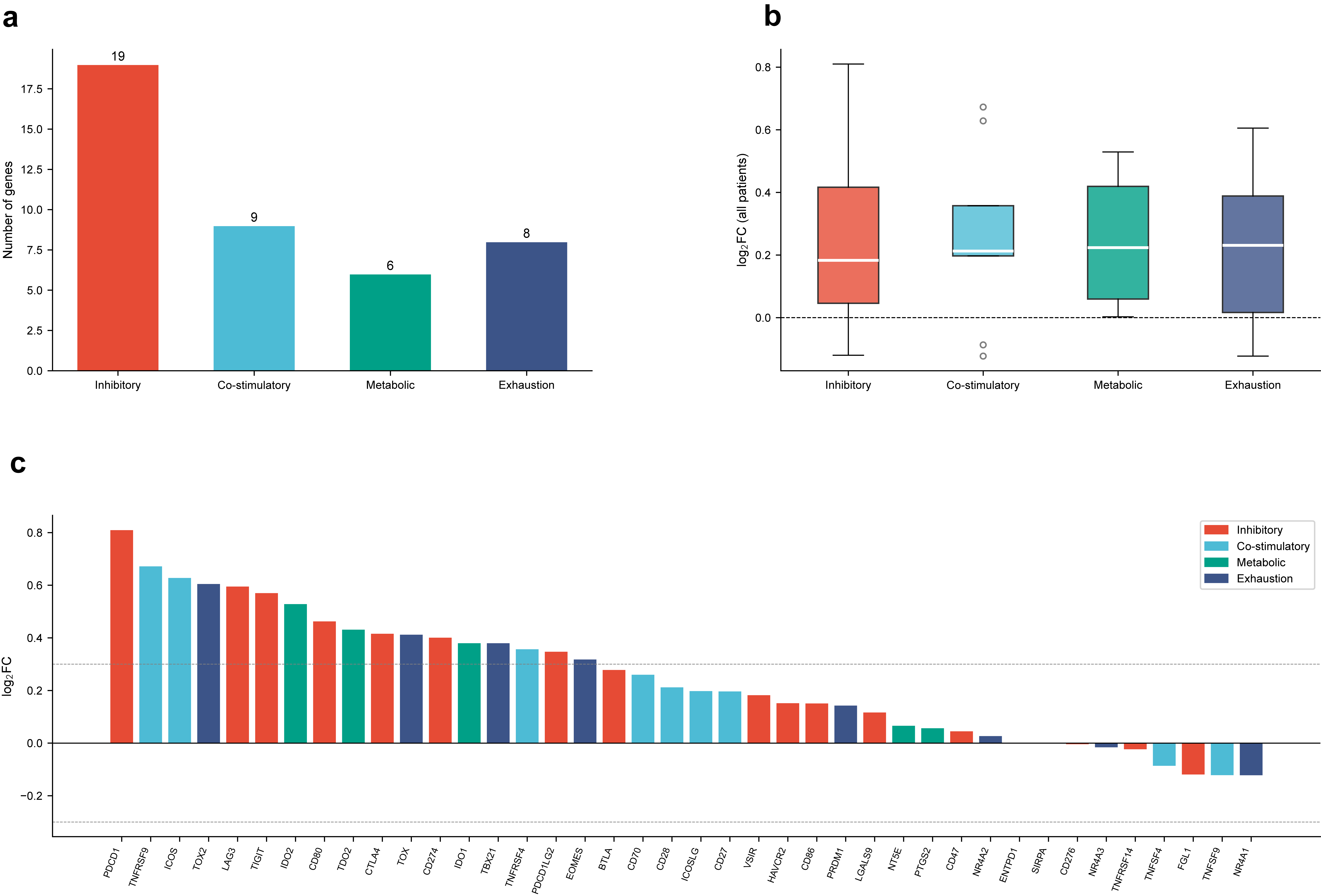

Supplement: Supplementary Figure 3 — Checkpoint Functional Categories. Bar charts and heatmaps stratified by functional category (inhibitory, co-stimulatory, metabolic, exhaustion). [file Image3.tif]

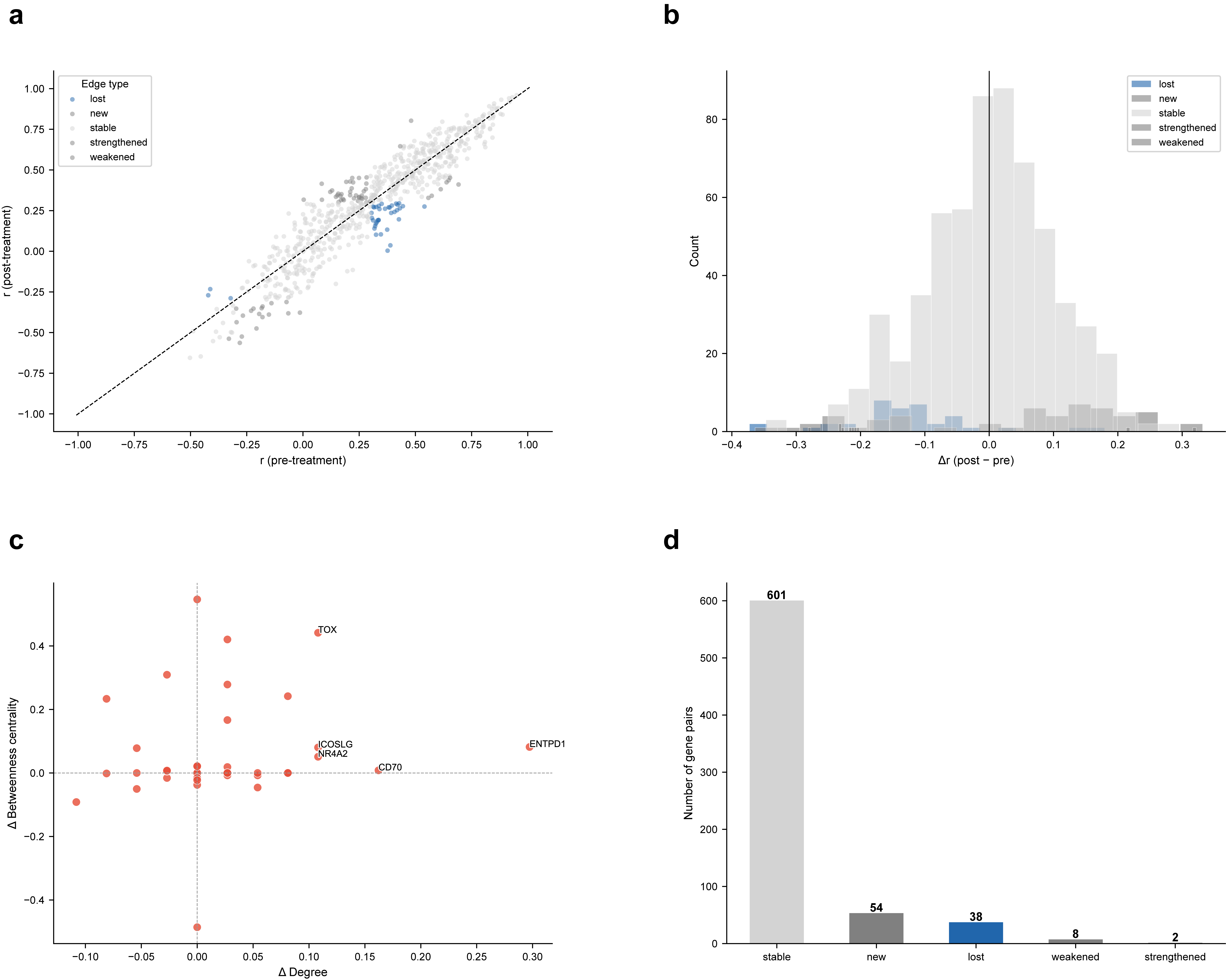

Supplement: Supplementary Figure 4 — Network Details. Edge-level network rewiring details; proportion of stable, new, and lost edges per gene pair. [file Image4.tif]

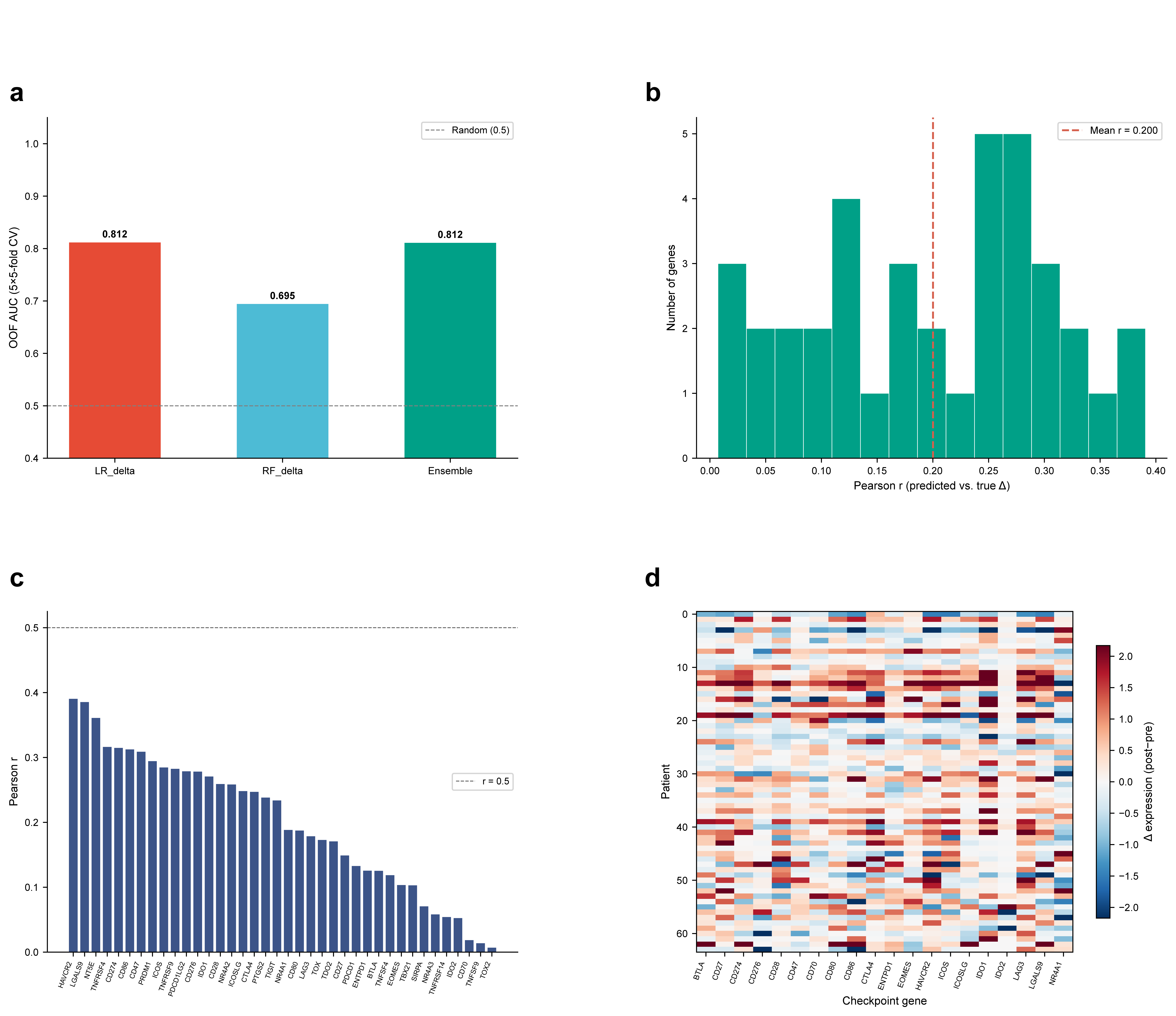

Supplement: Supplementary Figure 5 — Model Details. Individual model component performance (LR only, RF only); training loss and validation loss curves; fold-level AUC distribution across 5-fold × 5-repeat CV. [file Image5.tif]
